# OP37 How Can We Make Innovative Payment Schemes Work? Findings Of A European Union Horizon Europe Project

**DOI:** 10.1017/S0266462325100950

**Published:** 2025-12-29

**Authors:** Michael Drummond, Vittoria Ardito, Ludovico Cavallaro, Oriana Ciani

## Abstract

**Introduction:**

Innovative payment schemes have been widely proposed as a solution for dealing with uncertainties in cost and outcomes associated with the introduction of new health technologies. These schemes incorporate risk sharing, either by changing the method of paying for the health technology or by requesting more long-term evidence on outcomes. However, payers’ experiences with these schemes are mixed.

**Methods:**

A work package of the Health Innovation Next Generation Payment and Pricing Models (HI-PRIX) project is obtaining new evidence on the design and implementation of these schemes by: (i) undertaking a scoping review and classification of existing schemes, supplemented by interviews with national experts to identify schemes not in the published or gray literature; (ii) conducting a Delphi exercise with the key stakeholder groups involved in these schemes (payers, patient representatives, technology manufacturers, healthcare decision-makers), to identify the barriers and facilitators to developing schemes; and (iii) investigating several case studies to identify key learnings in the development of schemes.

**Results:**

To date, more than 200 innovative pricing schemes have been identified across a broad range of technologies and applications. In addition, a novel classification has been developed to enable policymakers to search for schemes meeting specific objectives or having particular characteristics. Also, a preliminary search of the database of schemes, the Pay-for-Innovation Observatory (hiprixhorizon.eu), suggests that it will be possible to identify whether schemes with certain characteristics are more likely to be successful when applied to certain types of technologies (e.g., cell and gene therapies, rare disease treatments, medical devices), or designed to meet particular objectives, including equity enhancing purposes.

**Conclusions:**

Despite the challenges, innovative payment schemes still represent one of the best approaches for facilitating the cost-effective introduction of new technologies under uncertainty. If policymakers can be assisted in selecting the best schemes to meet their particular objectives and are better informed of the main barriers and facilitators, then the success rate of these schemes will increase.

